# Hybridization between Yellowstone Cutthroat Trout and Rainbow Trout Alters the Expression of Muscle Growth-Related Genes and Their Relationships with Growth Patterns

**DOI:** 10.1371/journal.pone.0141373

**Published:** 2015-10-20

**Authors:** Carl O. Ostberg, Dorothy M. Chase, Lorenz Hauser

**Affiliations:** 1 U.S. Geological Survey, Western Fisheries Research Center, Seattle, Washington, United States of America; 2 School of Aquatic and Fishery Sciences, University of Washington, Seattle, Washington, United States of America; Universitat de Barcelona, SPAIN

## Abstract

Hybridization creates novel gene combinations that may generate important evolutionary novelty, but may also reduce existing adaptation by interrupting inherent biological processes, such as genotype-environment interactions. Hybridization often causes substantial change in patterns of gene expression, which, in turn, may cause phenotypic change. Rainbow trout (*Oncorhynchus mykiss*) and cutthroat trout (*O*. *clarkii*) produce viable hybrids in the wild, and introgressive hybridization with introduced rainbow trout is a major conservation concern for native cutthroat trout. The two species differ in body shape, which is likely an evolutionary adaptation to their native environments, and their hybrids tend to show intermediate morphology. The characterization of gene expression patterns may provide insights on the genetic basis of hybrid and parental morphologies, as well as on the ecological performance of hybrids in the wild. Here, we evaluated the expression of eight growth-related genes (MSTN-1a, MSTN-1b, MyoD1a, MyoD1b, MRF-4, IGF-1, IGF-2, and CAST-L) and the relationship of these genes with growth traits (length, weight, and condition factor) in six line crosses: both parental species, both reciprocal F1 hybrids, and both first-generation backcrosses (F1 x rainbow trout and F1 x cutthroat trout). Four of these genes were differentially expressed among rainbow, cutthroat, and their hybrids. Transcript abundance was significantly correlated with growth traits across the parent species, but not across hybrids. Our findings suggest that rainbow and cutthroat trout exhibit differences in muscle growth regulation, that transcriptional networks may be modified by hybridization, and that hybridization disrupts intrinsic relationships between gene expression and growth patterns that may be functionally important for phenotypic adaptations.

## Introduction

Hybridization creates a diversity of novel gene combinations that can have profound evolutionary consequences, including acting as a foundation for adaptive genetic variation, causing shifts in genetic traits that underlie phenotypic variation, and influencing hybrid fitness [1, 2]. Novel gene combinations may influence these evolutionary outcomes by disrupting transcriptional networks, which, in turn, may affect functional and regulatory mechanisms that control transcription, translation, and/or phenotype [3–5]. Such disruption could occur if parental species have evolved different transcriptional regulation mechanisms. When divergent regulatory elements are combined in a hybrid background, transcription may become disrupted [4], producing extreme expression phenotypes that lie outside the range found in parent populations [6]. These extreme phenotypes, referred to as transgressive, may provide diversity for rapid adaptation, or alternatively, be maladaptive [2, 7]. Novel gene combinations also affect the mode of transcription regulation inheritance. Hybrids display additive, dominant, and nonadditive (over- and underdominance) modes of inheritance for transcription regulation [8–10]. However, the same gene may show different modes of inheritance for transcription regulation within and between hybrid generations [9], indicating that inheritance patterns in hybrids are unpredictable [11]. Thus, hybridization may cause substantial changes in gene expression with wide-ranging evolutionary implications.

Hybridization also has the potential to reduce biodiversity through loss of distinct gene pools and genetic homogenization [12]. For example, widespread introduction of non-native rainbow trout (*Oncorhynchus mykiss*) into cutthroat trout (*O*. *clarkii*) habitats and consequent introgressive hybridization has resulted in loss of native cutthroat trout throughout their range [13, 14]. Rainbow and cutthroat trout are sister species and shared a common ancestor approximately 3 million years ago [15]. Because rainbow-cutthroat hybrids are viable and fertile, native cutthroat trout populations are often replaced by hybrid swarms following rainbow trout introductions, causing concern for cutthroat trout conservation [13].

Rainbow and cutthroat trout are morphologically divergent: rainbow trout are generally robust whereas cutthroat trout are typically more slender, and their hybrids tend to be morphologically intermediate [16–18]. These morphological differences have been associated with swimming performance, and higher sustained swimming activity of rainbow trout and hybrids might afford them a competitive advantage over cutthroat trout [16, 19]. Because muscle comprises the bulk of body mass and muscle growth represents change in length, weight, and morphology [20], the identification of mechanisms contributing to muscle growth patterns could provide insight into the underlying genetic basis of the difference between rainbow and cutthroat trout body shapes and its manifestation in their hybrids.

Muscle growth and development, myogenesis, is a highly integrated and complex process requiring a suite of gene products [21]. The myogenesis program involves specification of stem cells into myoblasts followed by the proliferation and terminal differentiation of myoblasts. Terminally differentiated myoblasts have two fates: they can either fuse with other myoblasts and form myotubes or fuse with existing myotubes, resulting in an increase in cell number (hyperplasia), or they can be absorbed into muscle fibers, resulting in an increase in cell size (hypertrophy). In vertebrates with indeterminate growth, such as salmonids, both pre- and post-natal muscle growth occurs through hyperplasia and hypertrophy. In addition, muscle growth is influenced by the countering effects of protein synthesis and degradation, and, ultimately, the expression of genes that regulate these processes [20]. Therefore, differences in body morphology between rainbow and cutthroat trout could be maintained, in part, through the regulation of muscle growth-related genes.

Our objectives were to characterize expression patterns of muscle growth-related genes and describe relationships between gene expression and growth patterns (as inferred by length, weight, and condition factor) among rainbow trout, cutthroat trout, and their hybrids. We produced F1 and backcross hybrids, in addition to parental crosses, using rainbow trout (Rbt) and Yellowstone cutthroat trout (*O*. *c*. *bouvieri*) (Yct), because body morphology has been well characterized in these species and their hybrids [17, 18]. We targeted eight muscle growth-related genes: two myostatin orthologs, MSTN-1a and MSTN-1b, both of which appear to negatively regulate muscle growth [22]; three myogenic regulatory factors, the paralogs MyoD1a and MyoD1b [23], and MRF-4, which are transcription factors that act at different stages within the myogenesis program [24, 25]; two insulin-like growth factors, IGF-1 and IGF-2, which stimulate protein synthesis within muscle as well as cellular proliferation and differentiation within the myogenesis program [26]; and calpastatin, CAST-L (long isoform), which plays a role in muscle protein turnover by inhibiting the calpain proteases that degrade muscle proteins [27]. We posit that the differences in body shape among Rbt and Yct is a consequence of evolutionary differences in muscle growth regulation between the species. Therefore, we expected to find differential expression of growth related-genes among Rbt, Yct, and their hybrids, and that these differences in expression would be associated with growth patterns.

## Methods

Our study was conducted in accordance with the Guide for the Care and Use of Laboratory Animals (www.nap.edu/catalog/12910.html) and the AVMA Guidelines for the Euthanasia of Animals (https://www.avma.org/KB/Policies/Pages/Euthanasia-Guidelines.aspx). The protocol was approved by the Institutional Animal Care and Use Committee of the University of Washington (protocol# 4153–02). Experimental fish were housed at Western Fisheries Research Center (WFRC), US Geological Survey, Seattle, WA, USA. This federal government research facility has a containment and effluent treatment system that prevents the escape of fish into the wild.

### Experimental cross design and sample collection

We generated six experimental crosses to evaluate the expression of growth-related genes and growth patterns among rainbow, cutthroat, and their hybrids. The six crosses consisted of: Rbt, first generation Rbt backcross (bc-Rbt; Rbt female x F1 male), reciprocal F1 hybrids (F1-Rbt; Rbt female x Yct male, and F1-Yct; Yct female x Rbt male), first generation Yct backcross (bc-Yct; Yct female x F1 male), and Yct. The F1 male parents used to generate the backcrosses were produced by crossing Rbt females and Yct males. Gametes were collected from male and female Rbt (Hayspur stock), Hayspur Hatchery, Idaho Department of Fish and Game (IDFG), male and female Yct from Henry’s Lake Fish Hatchery and Fish Management Station (IDFG), and male F1 Rbt-Yct developed at WFRC using the Rbt and Yct stocks described above. Fin tissue was collected from each parent. Because fertilization success varied among crosses (possibly due to due to reduced egg quality in Yct compared to Rbt), three families of Rbt, bc-Rbt, and F1-Rbt, and two families of Yct, bc-Yct, and F1-Yct developed.

After fertilization, each family was incubated in a randomly assigned Heath tray at 9°C and, at 55 days post-fertilization, transferred to a randomly assigned 275-L flow-through tank. Fish were culled at 93 days post-fertilization in order to equalize the number of fish across tanks (85 fish per tank). At this time, and during the course of the experiment, we applied the same feed ration to each tank following Westers’ recommended feeding regime [28]. At 119 days post-fertilization, each family within a cross was differentially marked with a specific fin clip, so that families could be combined within cross and be easily identified during sampling. Fish were anesthetized using 150 mg/L tricaine methanesulfonate (MS-222) prior to fin clipping. Families within cross were combined into randomly assigned 275-L flow through tanks and acclimated to 16°C.

At 145, 234, and 327 days post-fertilization we euthanized nine fish per cross using 250 mg/L of MS-222, recorded weight (g) and fork length (mm), and sampled white muscle from each fish. Muscle tissue was flash-frozen at -80°C until RNA extraction. Food was withheld 48 hours prior to each sampling day. Sampling was equalized among families within cross; three individuals per Rbt, bc-Rbt, and F1-Rbt family, and four and five individuals per Yct, bc-Yct, and F1-Yct family were sampled per time point. In total, 162 experimental fish were used (6 crosses x 3 time points x 9 fish/cross/time point).

### RNA extraction and cDNA synthesis

Total RNA was isolated from the nine individuals sampled per cross per time point (145, 234, and 327 days post-fertilization) using RNeasy kits (QIAGEN, Valencia, CA, USA) following the manufacture’s recommendations with in-column DNase treatment. RNA was eluted in 50 μl of RNase-free water, quantified, and stored at -80°C. cDNA synthesis was performed using 1 μg of RNA with 1X RQ1 Buffer (Promega, Madison, WI, USA), 1 unit RNasin (Promega), 0.25 units DNase I (Promega), and incubation at 37°C for 30 minutes followed by 70°C for 5 minutes. Next, 125 ng each of oligo (dt)15 and random hexamer primers (Promega) were added and annealed at 70°C for 5 minutes, followed by 2 minutes on ice. Finally, 1mM dNTP (Promega), 1.25X RT buffer (Promega), 1 unit RNasin (Promega), and 100 units of M-MLV reverse transcriptase (Promega) were added and incubated at 42°C for 60 minutes, followed by 70°C for 15 minutes. All cDNA was stored at -20°C.

### TaqMan assay design

Partial gene sequences for muscle growth-related genes were isolated from each parental fish (Rbt, Yct, and F1 hybrids) that was used to generate the experimental crosses in order to design TaqMan assays at gene sequences conserved between parental species. Designing TaqMan assays at the conserved gene regions eliminated inter-specific sequence variation as a source for differential estimates of gene expression. DNA from each parent was extracted from fin tissues using DNeasy kits (QIAGEN). We designed sequencing primers for eight muscle growth-related genes (target genes) and one reference gene (β-actin) using sequences obtained from GenBank (S1 Table) and Primer3 v.0.4.0 (http://frodo.wi.mit.edu/). Each parent was sequenced with each primer set. PCR amplifications were performed in 20 µl reaction volumes, consisting of 15 ng genomic DNA, 1X NH4 Reaction Buffer (Bioline, Taunton, MA, USA), 1.5–2.5 mM MgCl_2_, 200 μM each dNTP (Bioline), 150 nM of each primer, and 0.5 units Taq (Bioline). Cycling conditions consisted of 95°C for 2 minutes, followed by 35 cycles of 95°C for 15 seconds, anneal for 45 seconds (58–68°C, S1 Table), and 72°C for 1 minute. PCR products were sequenced using a 3730xl DNA Analyzer (Applied Biosystems, Carlsbad, CA, USA) and sequences were edited and aligned using SEQUENCHER v 4.10.1 (Gene Codes Corporation, Ann Arbor, MI, USA).

Gene regions conserved between Rbt and Yct parents were identified and TaqMan assays within these conserved regions were designed using Primer Express v 2.0.0 (Applied Biosystems) (S2 Table). Primer and probe sequences were subjected to BLAST analysis against the GenBank Rbt nucleotide collection to ensure homology to a single gene in the duplicated salmonid genome. Probe sequences had a minimum of three nucleotide mismatches with non-target Rbt genome sequences; with the exception of MSTN-1b in which the probe had two nucleotide mismatches with MSTN-1a (both the forward and reverse MSTN-1b primers also had two base mismatches with MSTN-1a).

### Real-time PCR and data analysis

Quantitative PCRs (qPCR) were performed using the ABI PRISM 7900HT Sequence Detection System (Applied Biosystems), applying standard cycling conditions consisting of 50°C for 2 minutes, 95°C at 10 minutes, followed by 40 cycles of 95°C for 15 seconds and 60°C for 1 minute. Reactions were performed in duplicate in 12 μl volumes containing 1X ABI Universal PCR Master Mix (Applied Biosystems), 900 nM forward primer, 900 nM reverse primers, 200 nM probe, and 5 μl total cDNA. Fluorescent threshold for the determination of threshold cycle (*C*_*T*_) values was set manually in the exponential phase of amplification. Internal controls were included with each run and consisted of no cDNA amplification controls (no reverse transcriptase or RNA added in reverse transcription reactions) and no template controls (water in place of cDNA template in qPCR).

The suitability of three potential reference genes, β-actin, acidic ribosomal phosphoprotein P0 (ARP), and 18s ribosomal RNA (18s) (S2 Table) was assessed using BestKeeper [29]. Across the 162 sample data set, *C*_*T*_ values were highly correlated between reference genes (ARP/β-actin, r = 0.77; ARP/18s, r = 0.80; and β-actin /18s, r = 0.73). We considered ARP and β-actin as the most stable reference genes.

Data were normalized by calculating *ΔC*_*T*_, the difference in *C*_*T*_ values between the target gene and the geometric mean for the reference genes. As transcript abundances have a negative logarithmic relationship with *ΔC*_*T*_ values, we estimated gene expression by applying $\frac{1}{Log(\Delta C_{T})}$. This method allowed the same transcript abundance estimate to be used for statistical testing within and among time points.

### Statistical analyses

We used weight, length, and condition factor, a weight-length relationship that describes relative body shape, as growth traits to compare differences in growth patterns among crosses. Condition factor was used as a proxy for body shape and was calculated as $K\;=\frac{W}{L^{3}}\times 100,000$ [30]; where K = condition factor, W = weight in grams, and L = fork length in millimeters. The PERMANOVA+ add-on package [31] in PRIMER v7 [32] was used to test for differences in weight, length, and condition factor among crosses within each time point. PERMANOVA+ is a non-parametric testing procedure that uses resemblance-based methods to allow the comparison among groups based on uni- and multivariate data and nested linear models. Pseudo-*F* statistics were tested for significance by 10,000 permutations under a reduced model and post-hoc tests for pairwise differences were also performed using PERMANOVA+. The model design consisted of cross as a fixed factor and family as a random factor nested within cross. Family was included as a factor to avoid pseudoreplication because data were not independent within families.

We used PERMANOVA+ to identify differences in transcript abundance among crosses within time point and within cross among time points following the method and model described above, with the exception that family was not nested in the within cross among time points tests, because the within cross model design allowed for testing of time point x family interactions. We removed the interaction from all tests where the interaction occurred at *P* < 0.05 and re-fit the model. A discriminant function analysis was used to summarize differences in transcript abundance across the eight genes among crosses within time point using the MASS package [33] in R v3.1.0 (R Development Core Team, www.R-project.org). Transcript abundance was standardized across all crosses within genes (i.e. was centered to a mean of zero and a variance of one) prior to discriminant function analysis. The contribution of each gene to the separation among crosses was evaluated by calculating the Spearman rank correlation coefficient (*r*_*s*_) between transcript abundance and canonical variates using *cor*.*test* in R.

To determine whether traits (weight, length, condition factor, and the percent Yct genome within individuals) accounted for differences in gene expression between parental species (Rbt and Yct grouped together) and hybrids (all hybrids grouped together), we tested for homogeneity of regression slopes using a non-parametric permutation-based analysis of covariance (ANCOVA). The percent Yct genome was defined as 0% in Rbt, 25% in bc-Rbt, 50% in both F1 hybrid crosses, 75% in bc-Yct, and 100% in Yct. We used gene expression as the response variable and trait as the covariate. ANCOVAs were performed using PERMANOVA+ with 10,000 permutations. Spearman’s rank correlation was used to explore how associations between transcript abundance and trait differed across parent species and across all hybrids.

The false discovery rate (B-Y FDR) procedure [34] was applied to correct for simultaneous tests and test results were considered as significant only when *P* exceeded the B-Y FDR adjusted critical value, *α*, for each series of simultaneous tests.

## Results

### Weight, length, and condition factor among crosses

**Fig 1:**
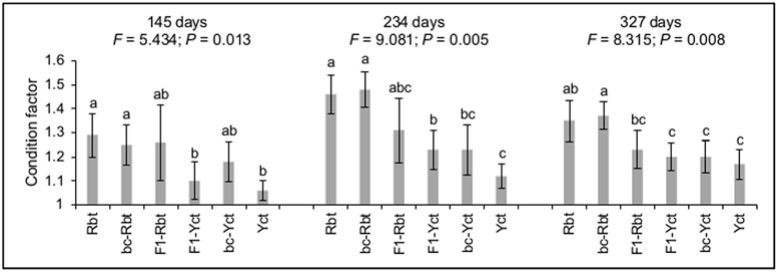
Mean condition factor (± SD) for each cross at each time point (145, 234, and 327 days post-fertilization). Results from PERMANOVA tests are shown (pseudo-*F* and *P*-value) and lowercase letters indicate significant differences (*P* < 0.05) in condition factor between crosses in post-hoc tests. Rbt = rainbow trout, bc-Rbt = first generation Rbt backcross (Rbt x F1), F1-Rbt = F1 hybrid with Rbt maternal lineage, F1-Yct = F1 hybrid with Yct maternal lineage, bc-Yct = first generation Yct backcross (Yct x F1), and Yct = Yellowstone cutthroat trout.

Weight and length were highly correlated across individuals (*r* = 0.947, *P* < 0.001), but neither length nor weight differed significantly among crosses within each time point following the B-Y FDR adjustment procedure for 9 simultaneous tests (critical value *α* = 0.0177) (S1 Fig). Condition factor differed among crosses at each time point (Fig 1). Rbt had higher condition factor than Yct within each time point. Condition factor differed between bc-Rbt and bc-Yct at 234 and 327 days post-fertilization, but neither backcross differed from their respective backcrossing parent. Family effects within cross were significant at 234 days (weight, pseudo-*F* = 4.101, *P* = 0.001; length, pseudo-*F* = 3.018, *P* = 0.008; condition factor, pseudo-*F* = 3.054, *P* = 0.007), but were non-significant at 145 and 327 days.

### Differences in gene expression among crosses

**Fig 2:**
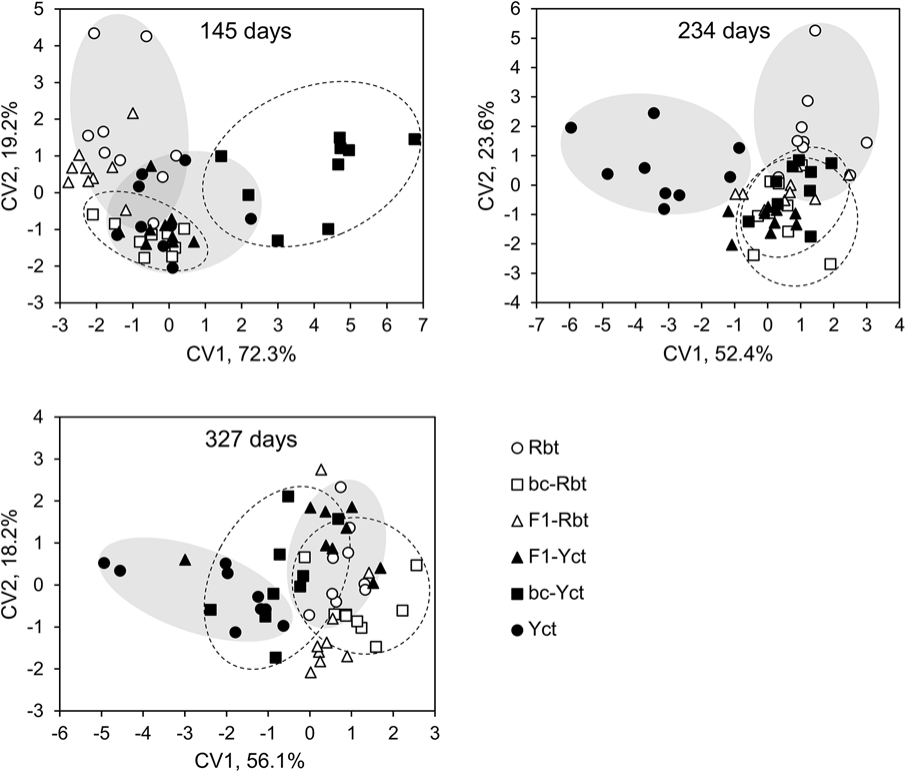
Canonical variates from the discriminant function analysis across eight muscle growth-related genes. Canonical variates for each individual and the percent variation on the first (CV1) and second axes (CV2) at 145, 234, and 327 days post-fertilization is shown. Rbt = rainbow trout, bc-Rbt = first generation Rbt backcross, F1-Rbt = F1 hybrid with Rbt maternal lineage, Yct = Yellowstone cutthroat trout, bc-Yct = first generation Yct backcross, and F1-Yct = F1 hybrid with Yct maternal lineage. Grey shaded ellipses indicate the spread of Rbt and Yct; stippled ellipses indicate the spread of bc-Rbt and bc-Yct.

More than 70% of the variation in transcript abundance across the eight genes was explained by the first and second canonical variates (CV) (Fig 2). The parental species were separated at 234 and 327 days, and marginally separated at 145 days. On the first canonical variate (CV1), hybrids were intermediate between parental species at 234 days, but appeared to be outside the range of parental species at other time points. On CV2, hybrids were intermediate between parental species at the earliest time point, but were outside the range of parental species at later time points. At 145 days post-fertilization, IGF-2 and MRF-4 contributed the most to the separation among crosses on CV1 and MyoD1b contributed the most to the separation on CV2 (Table 1). At 234 days, separation among crosses on CV1 was mainly influenced by CAST-L and the separation on CV2 was mostly influenced by IGF-2. At 327 days, MSTN-1a and MSTN-1b contributed the most to the separation on CV1 and the separation on CV2 was mostly influenced by CAST-L.

**Table 1:** Correlation between transcript abundance and the first (CV1) and second canonical variates (CV2) at 145, 234, and 327 days post-fertilization for eight muscle growth-related genes.

Day	Gene	CV1	CV2
**145**	MSTN-1a	0.140	-0.081
	MSTN-1b	0.361	0.130
	IGF-1	0.122	0.306
	IGF-2	0.489	-0.337
	MyoD1a	-0.027	0.550
	MyoD1b	-0.177	0.746
	MRF-4	0.491	0.586
	CAST-L	0.164	0.360
**234**	MSTN-1a	-0.466	-0.277
	MSTN-1b	-0.248	-0.249
	IGF-1	-0.092	-0.167
	IGF-2	-0.192	-0.354
	MyoD1a	-0.104	-0.129
	MyoD1b	-0.411	0.063
	MRF-4	0.051	0.225
	CAST-L	-0.674	-0.223
**327**	MSTN-1a	-0.729	0.542
	MSTN-1b	-0.710	0.409
	IGF-1	-0.025	0.079
	IGF-2	0.027	-0.041
	MyoD1a	0.058	0.649
	MyoD1b	-0.138	0.458
	MRF-4	0.296	0.290
	CAST-L	0.144	0.781

**Fig 3:**
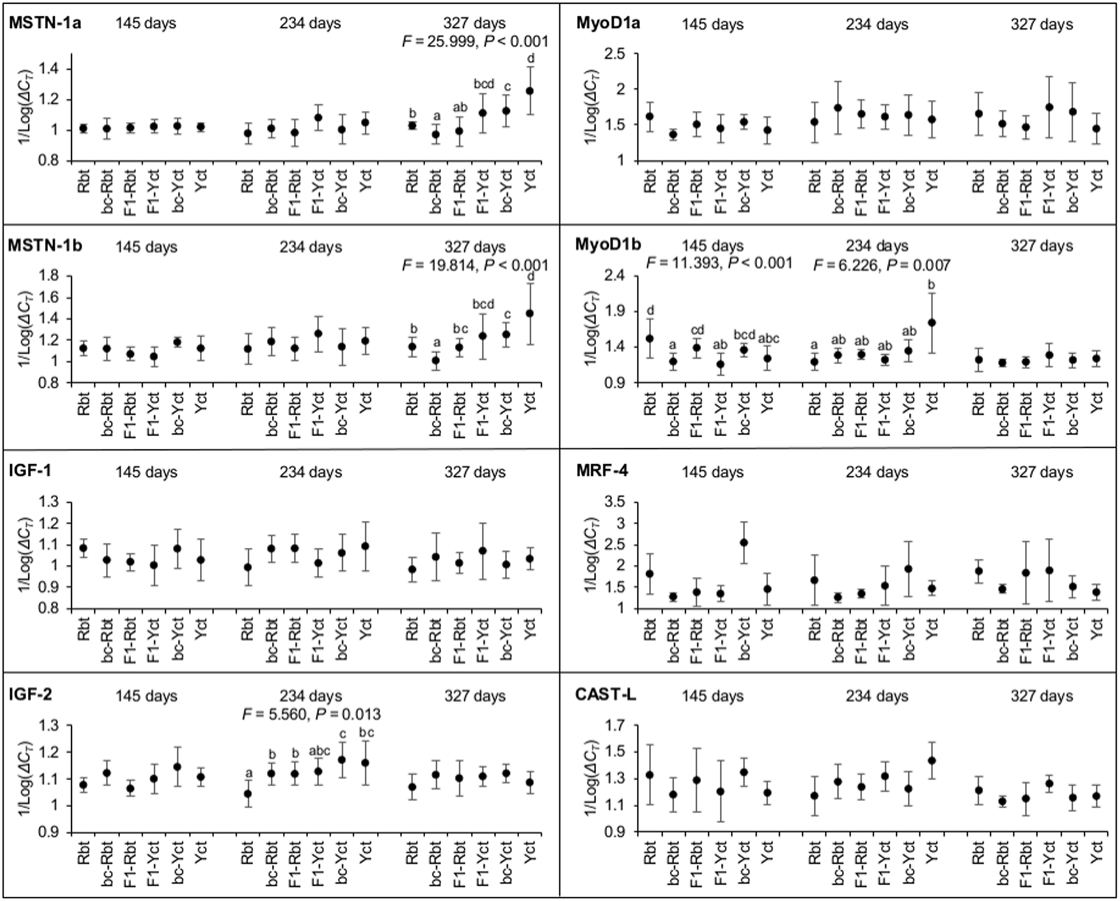
Transcript abundance (± SD) of eight muscle growth-related genes among crosses at 145, 234, and 327 days post-fertilization. Significant PERMANOVA tests are indicated (pseudo-*F* and *P*-value) and lowercase letters indicate significant differences (*P* < 0.05) in expression between crosses in post-hoc tests. Rbt = rainbow trout, bc-Rbt = first generation Rbt backcross, F1-Rbt = F1 hybrid with Rbt maternal lineage, Yct = Yellowstone cutthroat trout, bc-Yct = first generation Yct backcross, and F1-Yct = F1 hybrid with Yct maternal lineage.

Four genes were differentially expressed among crosses following the B-Y FDR adjustment procedure for 24 simultaneous tests (critical value *α* = 0.0132) (Fig 3). Family effects within cross were not significant. Parental species differed significantly in transcript abundance for MyoD1b at 145 days, IGF-2 and MyoD1b at 234 days, and for MSTN-1a and MSTN-1b at 327 days. Yellowstone cutthroat trout produced more transcripts than Rbt, with the exception of MyoD1b at 145 days. Genes that were differentially expressed between parental species tended to be expressed in hybrids at levels that were intermediate to the parental species, with the exception of MyoD1b, MSTN-1a and MSTN-1b in bc-Rbt, IGF-2 in bc-Yct, MyoD1b in F1-Yct, and MSTN1a in F1-Rbt. We found that bc-Rbt expressed fewer MSTN-1a and MSTN-1b transcripts than both parental species at 327 days. Transcript abundance differed between F1 hybrid crosses for MyoD1b at 145 days. Transcript abundance differed between backcrosses for MyoD1b at 145 days and for MSTN-1a and MSTN-1b at 327 days. Each backcross and their backcrossing parental species differentially expressed at least two genes.

### Difference in transcript abundance within cross among time points

Each cross differentially expressed at least one gene among time points, with the exception of F1-Yct, following the B-Y FDR adjustment procedure for 48 simultaneous tests (critical value *α* = 0.0112) (S2 Fig). Family effects within cross and time point x family interactions were not significant. Transcript abundance varied the most across time in Yct; MyoD1b and CAST-L expression peaked at 234 days and MSTN-1a and MSTN-1b expression peaked at 327 days. Rainbow trout differentially expressed IGF-1 and MyoD1b, and the expression of MyoD1b peaked earlier than in Yct. F1-Rbt differentially expressed MyoD1b only, and peak expression overlapped with both parental crosses. In each backcross, gene expression timing differed from their backcrossing parental species for two genes. Although MRF-4 expression levels in each parental species was not different across time points, transcript levels were higher in bc-Yct at 127 days and in bc-Rbt at 324 days. In addition, CAST-L transcript level peaked earlier in bc-Yct than in Yct and MyoD1a was differentially expressed among time points in bc-Rbt but not in Rbt.

### Relationships between transcript abundance and phenotypic traits

We found that traits accounted for differences in gene expression between parental species and hybrids. The ANCOVA revealed eight instances where regression slopes differed significantly between parental species and hybrids following the B-Y FDR adjustment for 92 simultaneous tests (*α* = 0.009) (Table 2). Correlations between transcript abundance and trait values were significant across parental species in six instances and significant across hybrids in two instances where regression slopes differed (Table 2). Across parental species, transcript abundance at three genes was significant and positively correlated with length, indicating that higher expression was associated with greater length. Two correlations between transcript abundance and percent Yct genome were significant, one was positive and one was negative. Transcript abundance at one gene was significant and negatively correlated with condition factor. Across hybrids, the two significant correlations involved the percent Yct genome only, and both were positive.

**Table 2:** Significant ANCOVAs (pseudo-*F* and *P*-value) and Spearman rank correlation coefficients (*r*_*s*_) between trait and transcript abundance across parent species and across hybrids within each time point.

Day	trait	gene	Pseudo-*F*	*P* (ANCOVA)	Parent *r*_*s*_	Hybrid *r*_*s*_
145	% YCT	MyoD1a	9.9641	0.002	-0.503	0.407*
145	% YCT	MyoD1b	10.582	0.002	-0.569*	0.329
234	% YCT	CASTL	7.7067	0.008	0.677*	-0.21
234	% YCT	MRF-4	12.324	<0.001	-0.015	0.471*
234	K	MyoD1b	13.01	<0.001	-0.651*	0.021
234	L	IGF1	14.253	0.001	0.754*	0
234	L	IGF2	16.203	<0.001	0.647*	-0.237
234	L	MyoD1b	17.183	<0.001	0.726*	-0.045

The traits are: percent Yct genome (% Yct), condition factor (K), length (L), and weight (W).

*Significant *r*_*s*_ following the false discovery rate procedure for 16 simultaneous tests (FDR adjusted *α* = 0.015).

## Discussion

Our study indicates that muscle growth-related genes were differentially expressed among Rbt, Yct, and their hybrids. Temporal trends in gene expression within crosses contributed to the differences among crosses, suggesting that the timing of gene expression differs among Rbt, Yct, and their hybrids during juvenile development. Consequently, different genes contributed to the variation among crosses over time. The expression of several genes was related to growth traits across parent species, but these relationships were not observed across hybrids, which could suggest that hybridization disturbed these relationships. Taken together, these results suggest that hybridization between Rbt and Yct disrupts gene regulation, thereby disturbing intrinsic relationships between gene expression and growth patterns that have evolved in each species.

### Growth patterns among crosses

Condition factor, but not length and weight, differed among crosses and appeared to be related to the proportion of Yct genome contained within cross. The differences in condition factor were consistent with relative body shapes expected for Rbt, Yct, and hybrid crosses [17, 18]. Considering that muscle comprises the bulk of body mass in fish [20], the difference in condition factor (this study) and morphology [17, 18] suggest that muscle growth differs among Rbt, Yct, and hybrids.

### Differential expression of muscle growth-related genes

Differential patterns of gene expression between Rbt and Yct could indicate inherent differences in regulatory pathways associated with myogenesis during juvenile development, providing a possible mechanism for their body shape differences. Modulation of gene expression is recognized as an important mechanism of phenotypic change [35–37]; therefore, variation in expression of muscle growth-related genes may be expected between Rbt and Yct. Below, we provide possible explanations of how muscle growth differed between parental species based on the functional roles of the genes investigated that were differentially expressed.

The elevated and coincident expression of IGF-2 and MyoD1b in Yct at 234 days could suggest that Yct had a higher rate of myogenesis at this time point, relative to Rbt. IGFs stimulate hypertrophic growth by activating transcriptional signaling pathways that regulate muscle fiber mass and size (reviewed in [21] and [26]). Specifically, muscle-derived IGF-2 mediates a signaling pathway that up-regulates MyoD expression [38]. MyoD specifies stem cells to enter the myogenic program, and stimulates the expression of genes downstream in the myogenic program that are required to initiate differentiation [38]. Although increased level of MyoD1b expression could indicate that Yct committed more cells to the myogenic program at 234 days, it does not imply that myoblasts differentiated and were incorporated into muscle. Differentiation involves myogenin, MRF-4, and genes of the MEF-2 family, and we found no difference in expression of MRF-4 between Yct and Rbt. However, the level of expression among these genes involved with differentiation have been found to differ across developmental stages in Rbt [39]. Therefore, it is possible that myogenin and/or MEF-2 family genes may have been expressed at higher levels in Yct, allowing myoblasts to differentiate and incorporate into muscle. Nevertheless, higher expression of IGF-2 and MyoD1b in Yct relative to Rbt could indicate a functional relationship among these genes that might be expected during periods of increased muscle growth.

Higher expression of MSTN-1a and MSTN-1b in Yct at 327 days could suggest that muscle growth declines earlier in Yct, than Rbt. Reduction of myostatin expression has been associated with faster growth [22, 40, 41] and muscle mass has been found to increase in the absence of functional myostatin [42–44]. The timing of myostatin expression during development in Rbt has been well characterized [39, 45]. Peak myostatin expression in Rbt occurs in 140 gram and larger fish [39], which is a similar size of our Rbt at 327 days. However, we did not find a temporal trend in myostatin expression in Rbt. In contrast, Yct at 327 days expressed both myostatins at higher levels than earlier time points, indicating that myostatin expression levels rise earlier in juvenile Yct development than in Rbt. Our data may suggest that the relatively slender body shape of Yct is due in part by early declines in muscle growth caused by myostatin expression.

Rainbow trout and Yct exhibited differential patterns in the timing of gene expression. Temporal differences in expression may suggest that these muscle growth-related genes affect different life stages during juvenile development between the species. Furthermore, the timing of gene expression in hybrids was often different from parental species, as well as among hybrids, suggesting that genes may affect hybrids and parental species at different life stages. In salmonids, hyperplasia and hypertrophy have been reported to make different contributions to muscle growth between populations that differ in growth rate [46, 47]. Taken together with the difference in condition factor (our study) and morphology [17, 18], the timing of expression of muscle growth-related genes may be expected to differ among Rbt, Yct, and their hybrids.

Gene expression in hybrids was highly variable and difficult to predict based on the gene expression levels in the parental species. Overall, gene expression in hybrids was roughly intermediate between that of Rbt and Yct. However, hybrids exhibited large variation in expression among genes, as well as temporally highly variable expression, and, occasionally, transgressive expression (MSTN-1a and MSTN-1b in bc-Rbt at 327 days). Hybrids often exhibit higher variation in gene expression than their parents [9–11, 48]. This variation is largely presumed to be a result of divergence in elements of transcriptional pathways between parental genomes, causing functional changes in gene regulation when these elements are inherited in their hybrids [4, 49]. Furthermore, transcription regulation is highly polygenic [6] and demonstrates complex patterns of inheritance that contribute to gene expression variation in hybrids [5]. For example, many studies comparing gene expression in hybrids to their parental types have found that nonadditive transcription regulation inheritance is fairly common in hybrids [8–10, 50]. These nonadditive gene interactions promote high variability. Additionally, because recombination between parental genomes may break down co-adapted transcriptional pathways and create novel gene combinations, gene expression may be expected to be more variable in backcross hybrids than F1 hybrids. Our data, albeit limited, support this hypothesis, which is consistent with findings in lake whitefish (*Coregonus clupeaformis*) [9].

### Relationships between transcript abundance and phenotypic traits

Significant relationships between transcript abundance and growth traits observed across the parental species were not conserved across hybrids, indicating that hybridization disturbed gene expression-growth trait relationships. Such associations between gene expression and growth traits could indicate functional impacts of hybridization between Rbt and Yct populations. These relationships, limited to length and condition factor, suggest that hybridization may alter co-adapted transcriptional networks associated with fitness-related traits. Although the relationships were correlational, rather than causal, they indicate associations between transcript abundance and growth, and suggest a mechanistic link between gene expression and phenotype. Furthermore, gene expression-growth trait relationships were not conserved across time points, indicating these relationships were dependent on size and/or age. Nevertheless, the irregularity of conserved gene expression-growth trait relationships across hybrids suggests that hybridization with Rbt could disrupt co-adapted functional genes within native Yct.

### Implications

From the perspective of cutthroat trout conservation, these results suggest that transcriptional signaling pathways inherently differ between Rbt and Yct and that hybridization with non-native Rbt may alter transcriptional regulation of genes that are functionally important for muscle growth. Consequently, difference in muscle growth regulation between rainbow and cutthroat trout could play a role in the hybridization dynamics that have been observed in admixed populations in the wild. Here, we generalize the discussion of our results with respect to hybridization studies between Rbt and westslope cutthroat trout (*O*. *c*. *lewisi*), coastal cutthroat trout (*O*. *c*. *clarki*) and Yct. Research on wild populations suggests that growth rate is positively associated with Rbt admixture [51–53]. Similar findings have been reported in a laboratory study [17]. Such differences in growth rate may be expected when parental species differ in muscle growth regulation. Research on wild populations also suggests that Rbt have higher metabolic demand than cutthroat trout [54], consistent with their higher sustained swimming activity, higher foraging rates and higher level of aggression [16, 18, 19, 55]. It is possible that the difference in metabolic demand and, therefore, swimming capacity, is a consequence of differences in muscle growth and development between Rbt and cutthroat trout. Finally, research on wild populations suggests that habitat is partitioned among parental species and their hybrids; Rbt occupy warmer habitats, cutthroat trout colder habitats, and hybrids intermediate habitats [53, 56]. Such habitat partitioning may be influenced by metabolic demands, food productivity and water temperatures that are required for optimum muscle growth and performance, depending on the level of admixture. Differences in muscle growth regulation could provide cutthroat trout with an advantage in cold streams with low productivity, while Rbt and hybrids may be more adapted to warmer streams that are more productive. Nevertheless, our results suggest that hybrids are not always intermediate between the two parental species, and that the ecological outcome of introgression may not only depend on extrinsic environmental factors, but also on intrinsic genetic changes that are not always predictable from simple estimates of percent introgression.

To conclude, our study provides evidence for muscle growth regulation differences between Rbt and Yct, and demonstrated that the expression of muscle growth-related genes and their relationship with growth traits is altered following hybridization. In addition, our results expand the current understanding of hybridization between Rbt and cutthroat trout; transcriptional networks may be modified in hybrids, resulting in unpredictable gene expression patterns. The growth-related genes herein may have application to future hybridization studies and could be used, for example, to characterize allelic expression patterns and identify relationships between these patterns and growth traits, as well as characterize relationships between gene expression and morphology within hybrid populations.

## Supporting Information

S1 Fig Mean length (mm), weight (g), and condition factor (one standard deviation indicated) for each cross at each time point (days post-fertilization). Results from PERMANOVA tests are shown (pseudo-*F* and *P*-value) and lowercase letters indicate significant differences (*P* < 0.05) between crosses in post-hoc tests. Rbt = rainbow trout, bc-Rbt = first generation Rbt backcross (Rbt x F1), F1-Rbt = F1 hybrid with Rbt maternal lineage, F1-Yct = F1 hybrid with Yct maternal lineage, bc-Yct = first generation Yct backcross (Yct x F1), and Yct = Yellowstone cutthroat trout.
(PDF)

S2 Fig Transcript abundance (± SD) of eight muscle growth-related genes within cross among time points (145, 234, and 327 days post-fertilization). Significant PERMANOVA tests are indicated (pseudo-*F* and *P*-value) and lowercase letters indicate significant differences (*P* < 0.05) among days within cross.
(PDF)

S1 Table PCR primers, and anneal temperature, used to isolate partial gene sequences from parental fish. (PDF)

S2 Table Primer and probes used for real-time PCR. (PDF)
