# Supplementary material for: Hybridization between Yellowstone Cutthroat Trout and Rainbow Trout Alters the Expression of Muscle Growth-Related Genes and Their Relationships with Growth Patterns
Source: PLoS One. 2015 Oct 20;10(10):e0141373. doi: 10.1371/journal.pone.0141373 (PMC4612777; doi:10.1371/journal.pone.0141373)

S1 Fig. **Mean length (mm), weight (g), and condition factor (one standard deviation indicated) for each cross at each time point (days post-fertilization).** Results from PERMANOVA tests are shown (pseudo-*F* and *P*-value) and lowercase letters indicate significant differences ($P < 0.05$) between crosses in post-hoc tests. Rbt = rainbow trout, bc-Rbt = first generation Rbt backcross (Rbt x F1), F1-Rbt = F1 hybrid with Rbt maternal lineage, F1-Yct = F1 hybrid with Yct maternal lineage, bc-Yct = first generation Yct backcross (Yct x F1), and Yct = Yellowstone cutthroat trout.

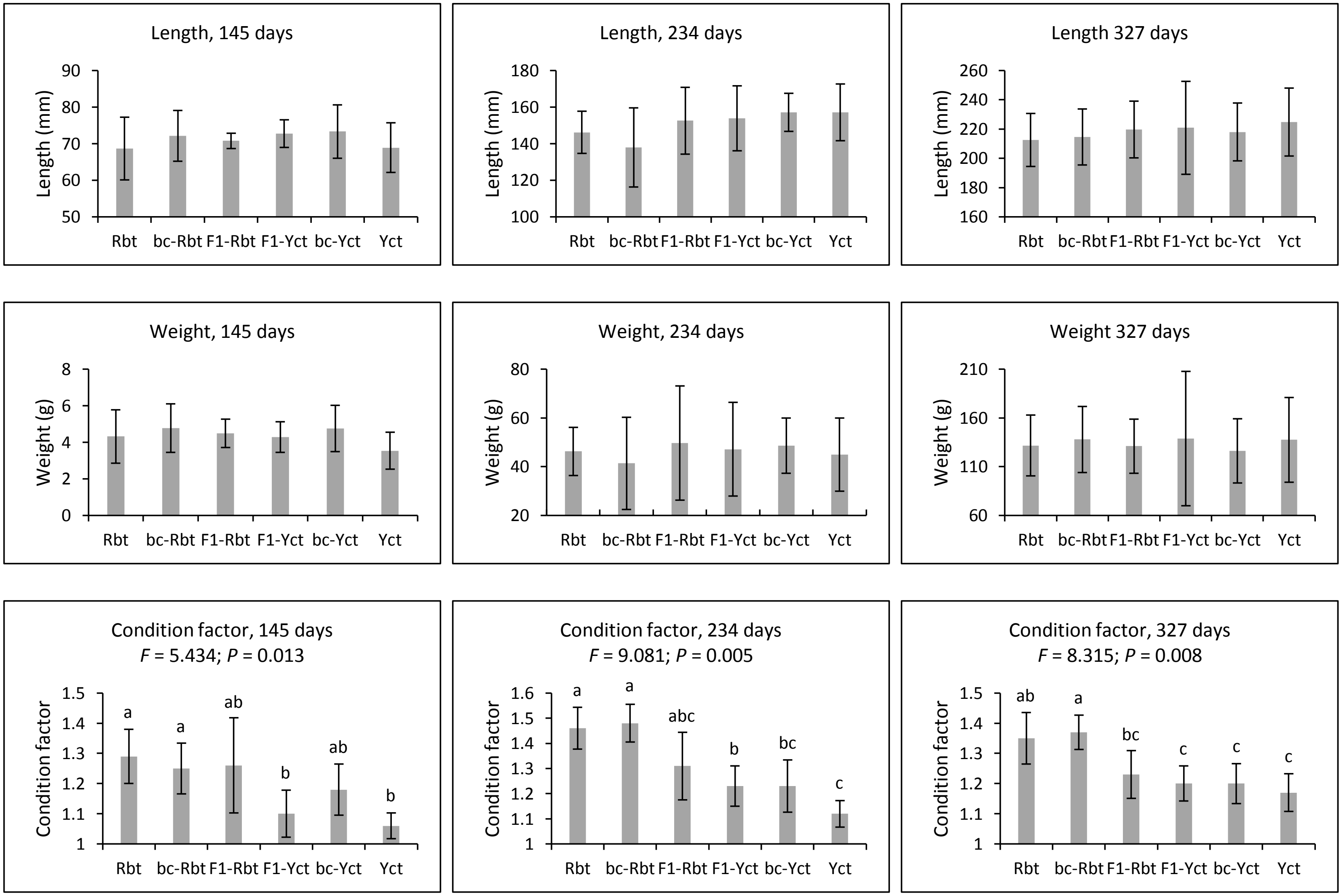

Supplement: S1 Fig — Results from PERMANOVA tests are shown (pseudo-F and P-value) and lowercase letters indicate significant differences (P < 0.05) between crosses in post-hoc tests. Rbt = rainbow trout, bc-Rbt = first generation Rbt backcross (Rbt x F1), F1-Rbt = F1 hybrid with Rbt maternal lineage, F1-Yct = F1 hybrid with Yct maternal lineage, bc-Yct = first generation Yct backcross (Yct x F1), and Yct = Yellowstone cutthroat trout. (PDF) [file pone.0141373.s001.pdf]
