# Supplementary material for: Hybridization between Yellowstone Cutthroat Trout and Rainbow Trout Alters the Expression of Muscle Growth-Related Genes and Their Relationships with Growth Patterns
Source: PLoS One. 2015 Oct 20;10(10):e0141373. doi: 10.1371/journal.pone.0141373 (PMC4612777; doi:10.1371/journal.pone.0141373)
Supplement: S2 Fig — Significant PERMANOVA tests are indicated (pseudo-F and P-value) and lowercase letters indicate significant differences (P < 0.05) among days within cross. (PDF) [file pone.0141373.s002.pdf]

S2 Fig. Transcript abundance (± SD) of eight muscle growth-related genes within cross among time points (145, 234, and 327 days post-fertilization). Significant PERMANOVA tests are indicated (pseudo-*F* and *P*-value) and lowercase letters indicate significant differences ($P < 0.05$) among days within cross.

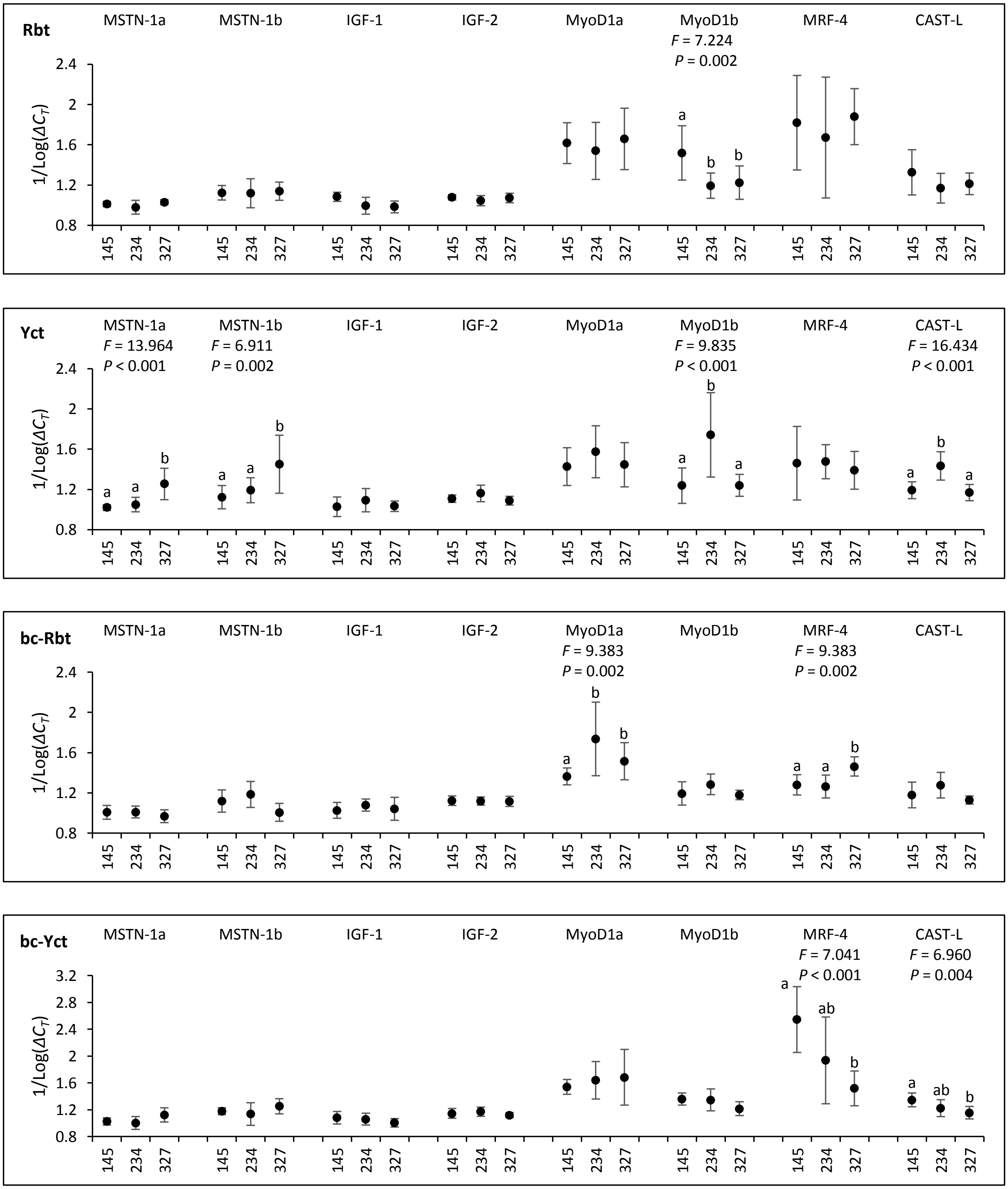

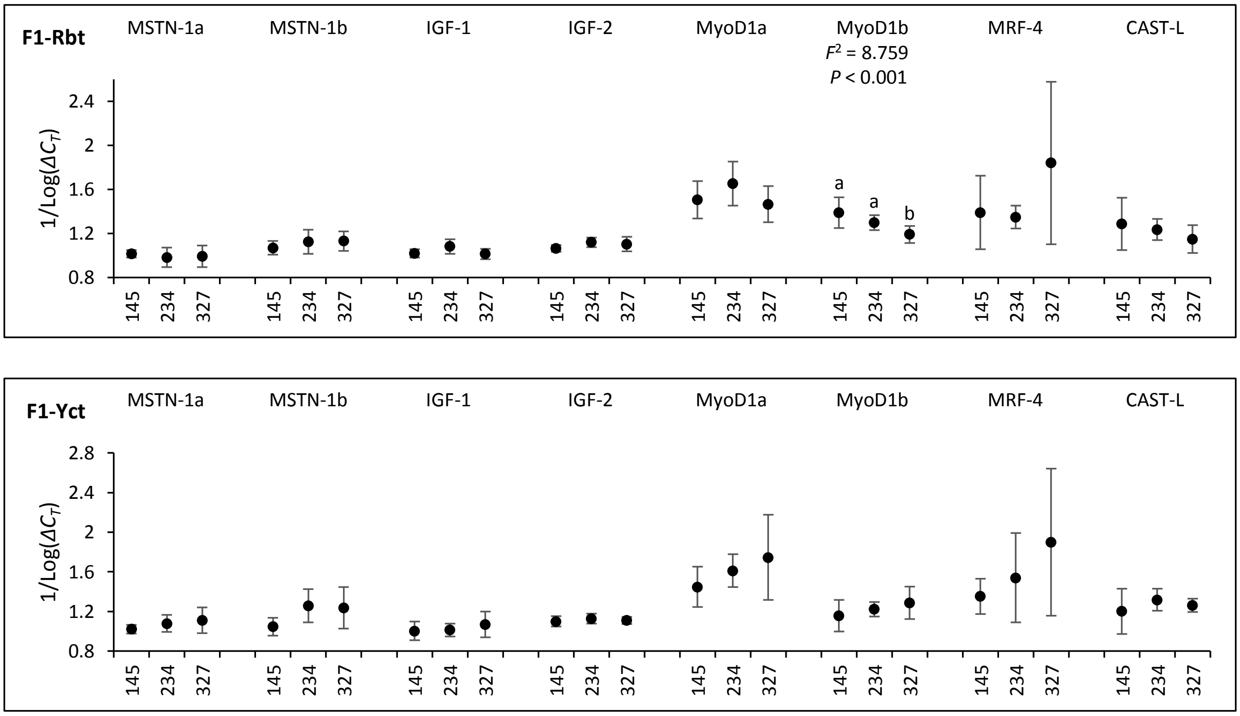
F1-Rbt
MSTN-1a
MSTN-1b
IGF-1
IGF-2
MyoD1a
MyoD1b
F^2 = 8.759
P < 0.001
MRF-4
CAST-L
1/Log(ΔC_T)
a
a
b
145
234
327
F1-Yct
MSTN-1a
MSTN-1b
IGF-1
IGF-2
MyoD1a
MyoD1b
MRF-4
CAST-L
1/Log(ΔC_T)
145
234
327
