# Supplementary material for: Hybridization between Yellowstone Cutthroat Trout and Rainbow Trout Alters the Expression of Muscle Growth-Related Genes and Their Relationships with Growth Patterns
Source: PLoS One. 2015 Oct 20;10(10):e0141373. doi: 10.1371/journal.pone.0141373 (PMC4612777; doi:10.1371/journal.pone.0141373)
Supplement: S1 Table — (PDF) [file pone.0141373.s003.pdf]

S1 Table. PCR primers, and anneal temperature, used to isolate partial gene sequences from parental fish.

Gene	Primer sequence
IGF-1 (68 ºC)	Forward: 5'-AGAACAAACTGGAACGACAGC-3'
AF063216	Reverse: 5'-TGTCCATGCTGTTTCACTGAT-3'
IGF-2 (68 ºC)	Forward: 5'-AGTGGGCCAATTGATCTGTC-3'
X97225	Reverse: 5'-ACGGAGACAAGGGAGATGTG-3'
MSTN-1a (68 ºC)	Forward: 5'-CTCTGTAGTCCGCCTTCACAT-3'
DQ136028	Reverse: 5'-TGGCCATTGTCATGATTGTT-3'
MSTN-1b (66 ºC)	Forward: 5'-CCGCATCCGTAATTTGATTT-3'
DQ138300	Reverse: 5'-GCTTGACAACATCTCGGCTA-3'
MyoD1a (64 ºC)	Forward: 5'-GGAGTTGCCGGATATTCCTT-3'
AJ618978	Reverse: 5'-GGAATCATTTGGTGCTTCGT-3'
MyoD1b (66 ºC)	Forward: 5'-CGCAAGACGAAGCAACTATG-3'
FJ793566	Reverse: 5'-CCACAGCAACATTCTGCAAC-3'
MRF-4 (64 ºC)	Forward: 5'-TGAAGGCGTCCAATAAGGAG-3'
EF450079	Reverse: 5'-TCTCTTGAGGATTTGTTTCTGC-3'
CAST-L (58 ºC)	Forward: 5'-TCAAGTGGCCTGACTGACTG-3'
AY937407	Reverse: 5'-CTCACAGATGCATCCATATCAAG-3'
β-actin (64 ºC)	Forward: 5'-CATGTTTGAGACCTTCAACACC-3'
AF254414	Reverse: 5'-TGATCTTGATCTTCATGGTGGA-3'

GenBank accession number is provided below each gene.
