# Supplementary material for: Hybridization between Yellowstone Cutthroat Trout and Rainbow Trout Alters the Expression of Muscle Growth-Related Genes and Their Relationships with Growth Patterns
Source: PLoS One. 2015 Oct 20;10(10):e0141373. doi: 10.1371/journal.pone.0141373 (PMC4612777; doi:10.1371/journal.pone.0141373)
Supplement: S2 Table — (PDF) [file pone.0141373.s004.pdf]

S2 Table. Primer and probes used for real-time PCR.

Gene	Primer and probe sequence
IGF-1	Forward: 5'-TGTGCCTCTGTCCACGCTTT-3'
KC609005,KC609006	Reverse: 5'-AATGTACTGTGCCCCTGTCAAGTC-3'
	Probe: 6-FAM-TAGGTGTTCTTGGCATGTCTGTGTGGCG-TAMRA
IGF-2	Forward: 5'-CGCTCCCAGAACCGTGGTAT-3'
KC609007, KC609008	Reverse: 5'-TCTGTAGAGAGGTGGCCGACAC-3'
	Probe: 6-FAM-TGGAGGAGTGTTGTTTCCGTAGCTGTGACCT-TAMRA
MSTN-1a	Forward: 5'-ACACGCCATCAAGTCCCAAAT-3'
KC609015, KC609016	Reverse: 5'-CTGGTCAAGAAGTTGCTGCAAAG-3'
	Probe: 6-FAM-AGCAGAGATGTTGTCAAGCAGCTCCTGCCTAA-TAMRA
MSTN-1b	Forward: 5'-CCGCACCTTAGATAATGAATCTGATG-3'
KC609017, KC609018	Reverse: 5'-TTGGTGGTGCGCCGTTT-3'
	Probe: 6-FAM-TGAGTTTTATGGTTGCTTTCGGTCCAATGGG-TAMRA
MyoD1a	Forward: 5'-TCCTGCGGAATGCCATCAG-3'
KC609013, KC609014	Reverse: 5'-GGACTGGACGCATCCGAGTC-3'
	Probe: 6-FAM-ATTGAGTCTCTCCAAGGCCTGCTTCGTGG-TAMRA
MyoD1b	Forward: 5'-CCAGCCTGGATTGTCTTTCCA-3'
KC609011, KC609012	Reverse: 5'-CCCTCCTGGCCTGATAACACA-3'
	Probe: 6-FAM-TCGTGGAGAGAATCTCTACAGACACGTCAGCG-TAMRA
MRF4	Forward: 5'-TCGCCTGTCATCAATCGTTGA-3'
KC609009, KC609010	Reverse: 5'-GCTGACGTCGCTTATACAGGCTAC-3'
	Probe: 6-FAM-AAGAGAAACCGACTTGCAACGAAGAAGTCTCAG-TAMRA
CAST-L	Forward: 5'-AGAAAACTCACTCTTCTGTGTACAACAAT-3'
KC609003, KC609004	Reverse: 5'-GGGTAATAGTGAGTGTCATGTGTTTAGG-3'<br>Probe: 6-FAM-ACAGACGTCCCAATCCTACATAACCCCTATATACTGT-TAMRA
β-actin	Forward: 5'-GGAGAAGCTGTGCTACGTGGC-3'
KC888023, KC888024	Reverse: 5'-CTCGTTGCCGATGGTGATGA-3'
	Probe: 6-FAM-CTGGACTTTGAGCAGGAGATGGGCACC-TAMRA
ARP[1]	Forward: 5'-GAAAATCATCCAATTGCTGGATG-3'
Purcell et al. (2004)	Reverse: 5'-CCTCCCACGCAAGGACAGA-3'
	Probe: 6-FAM-CTATCCCAAATGTTTCATTGTCGGCGC-TAMRA
18s	Life Technologies catalogue number 4308329
X03205	

GenBank accession number or Taqman assay reference is provided below each gene.

[1]Purcell MK, Kurath G, Garver KA, Herwig RP, Winton JR. Quantitative expression profiling of immune response genes in rainbow trout following infectious haematopoletic necrosis virus (IHNV) infection or DNA vaccination. Fish & Shellfish Immunology. 2004; 17(5): 447-62.
